# The impact of population-based disease management services for selected chronic conditions: the Costs to Australian Private Insurance - Coaching Health (CAPICHe) study protocol

**DOI:** 10.1186/1471-2458-12-114

**Published:** 2012-02-10

**Authors:** Joshua M Byrnes, Stan Goldstein, Benjamin Venator, Christine Pollicino, Shu-Kay Ng, David Veroff, Christine Bennett, Paul A Scuffham

**Affiliations:** 1 Centre for Applied Health Economics, School of Medicine, Logan Campus, Griffith Health Institute, Griffith University, University Drive, Meadowbrook, Queensland 4131, Australia; 2 School of Public Health and Community Medicine, University of New South Wales, Anzac Pde, Kensington, NSW 2031, Australia; 3 Bupa Health Dialog, 801 Glenferrie Road, Hawthorn, VIC 3122, Australia; 4 Health Dialog, 60 State Street, Suite 1100, Boston, MA 02109, USA; 5 School of Medicine Sydney, University of Notre Dame, 160 Oxford St, Darlinghurst, NSW 2010, Australia; 6 Bupa Health Foundation, 201 Kent St, Sydney, NSW 2000, Australia

## Abstract

**Background:**

Recent evidence from a large scale trial conducted in the United States indicates that enhancing shared decision-making and improving knowledge, self-management, and provider communication skills to at-risk patients can reduce health costs and utilisation of healthcare resources. Although this trial has provided a significant advancement in the evidence base for disease management programs it is still left for such results to be replicated and/or generalised for populations in other countries and other healthcare environments. This trial responds to the limited analyses on the effectiveness of providing chronic disease management services through telephone health coaching in Australia. The size of this trial and it's assessment of cost utility with respect to potentially preventable hospitalisations adds significantly to the body of knowledge to support policy and investment decisions in Australia as well as to the international debate regarding the effect of disease management programs on financial outcomes.

**Methods:**

Intention to treat study applying a prospective randomised design comparing usual care with extensive outreach to encourage use of telephone health coaching for those people identified from a risk scoring algorithm as having a higher likelihood of future health costs. The trial population has been limited to people with one or more of the following selected chronic conditions: namely, low back pain, diabetes, coronary artery disease, heart failure, and chronic obstructive pulmonary disease. This trial will enrol at least 64,835 sourced from the approximately 3 million Bupa Australia private health insured members located across Australia. The primary outcome will be the total (non-maternity) cost per member as reported to the private health insurer (i.e. charged to the insurer) 12 months following entry into the trial for each person. Study recruitment will be completed in early 2012 and the results will be available in late 2013.

**Discussion:**

If positive, CAPICHe will represent a potentially cost-effective strategy to improve health outcomes in higher risk individuals with a chronic condition, in a private health insurance setting.

**Trial Registration:**

Australian New Zealand Clinical Trials Registry reference: ACTRN12611000580976

## Background

As growth in the prevalence of chronic disease increases the economic burdens on the health system and on society more broadly, approaches to ameliorating this impact have received increasing attention. Advance in medical science and management of both acute and chronic conditions have led to greater proportions of the population in many countries reaching older age. The impact on healthcare delivery systems has been compounded by lifestyle issues brought about by, for example, changes in diet, food availability, and activity levels. These changes have led to increasing financial pressures on healthcare systems to cope with the increased demand for health services [1-3].

Disease management - a term used to describe a wide range of approaches designed to mitigate the progression and quality of life impacts of health conditions and encourage adherence to recommended treatment plans and self-care strategies [4] - has been promoted as a way to improve quality of care, improve health outcomes and lower costs, particularly for patients with chronic disease [5-7].

Faced with double-digit healthcare inflation, disease management has been widely used in the United States by insurers, employers, and government with revenues to provider organisations reported in 2007 to be approaching $2 billion a year [8]. In addition, public health care funders in the United States have also considered further investment in disease management as a strategy to reduce health care expenditure [9]. However, in a review conducted in 2007, the evidence for such large scale investment into disease management programs to have a significant impact on financial outcomes was labelled 'inconclusive'. Beneficial claims of disease management programs have been criticised on the grounds that they have generally been anecdotal, involve highly selected patients in closed systems of care, or suffer from a number of biases, such as bias in the recruitment or enrolment of participants [10].

To address the apparent lack of large-scale, methodologically rigorous investigations into the effects of disease management on financial outcomes, a randomised trial of telephone care-management within the United States was conducted for a study population of 174,120 subjects [11]. The evidence from this large scale trial indicated that enhancing shared decision-making and improving knowledge, self-management, and provider communication skills to at-risk patients can reduce health costs and utilisation of healthcare resources.

Although this trial has provided a significant advance in the evidence base for disease management programs it remains to be seen whether such results can be replicated and/or generalised for populations in other countries or other health environments. For example, although the Australian health care system has a great many similarities to that in the United States, there remain significant differences in health financing, medical practice, and population risk differences. As such, it is yet to be shown whether the telephonic health coaching models that were effective in trials conducted within the United States [11] will confer similar benefits within the models of healthcare operating in Australia.

To date there have been few clinical trials of health coaching or disease management programs in Australia [1], and none of a similar scale or depth as that collected within the United States. Moreover, trials that have been conducted in Australia have generally only taken a public health sector perspective, whereas health care in Australia is provided both through public and private health care providers and financed through both private and public insurance schemes.

Although results arising from the public health sector may be relevant to the private health sector, there remain significant points of differentiation, such as case mix, population demographics, financing, terms of employment and waiting times [12-16].

The CAPICHe trial is a prospective study of at least 64,835 subjects, that will assess differences in costs and hospital utilisation for a cohort actively offered a disease management (health coaching) intervention compared with a control group of persons who will be advised that they can contact the coaching service and receive advice if they so wish. This trial responds to the limited large-scale analyses on the effectiveness of providing chronic disease management services through telephone health coaching in Australia. Furthermore, this trial provides data from the perspective of private health insurers, an analysis that is often absent in the Australian literature. Moreover, the size of this trial and its assessment of cost utility with respect to potentially preventable hospitalisations adds significantly to the body of knowledge to support policy and investment decisions in Australia as well as to the international debate regarding the effect of disease management programs on financial outcomes. This article outlines the design and analysis plan for the CAPICHe trial.

### Specific aims

The objective of the study is to evaluate the relative impact on healthcare utilisation and cost of participants in a disease management program provided through telephonic health coaching support versus usual care.

### Trial Design and Methods

**Figure 1:**
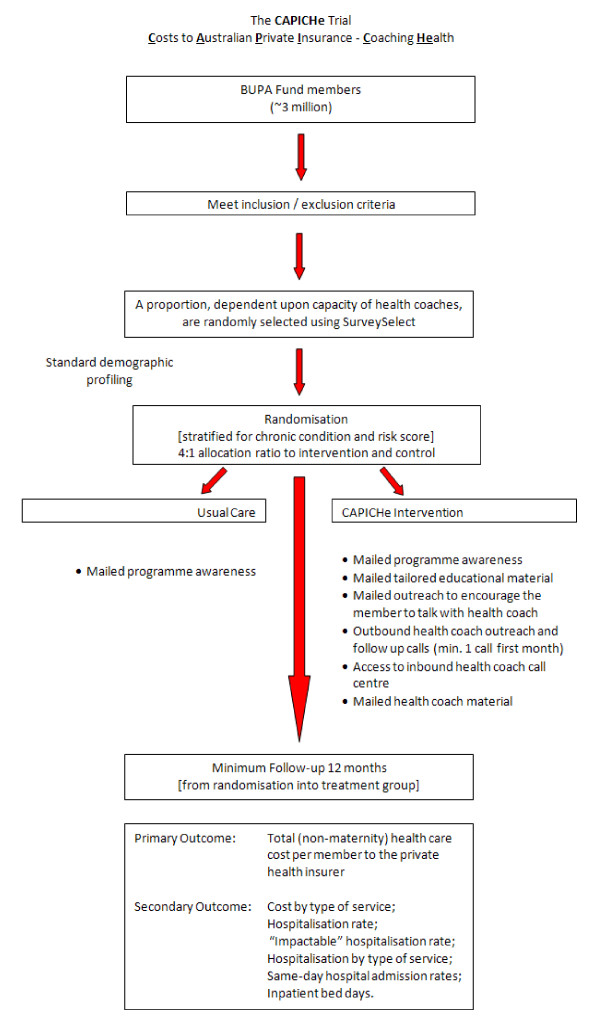
**Study design for CAPICHe**.

This is an intention to treat study applying a prospective randomised design comparing usual care with extensive outreach to encourage use of telephone health coaching. The population has been targeted prior to randomisation as people identified on the basis of previous medical and utilisation history using a risk scoring algorithm. The risk targeting presumes to pre-select people believed to have a high likelihood of further hospitalisation and health costs in the short term, based on previous analysis of population utilisation trends. The trial is limited to people who have had a diagnosis of one or more selected chronic conditions: namely, low back pain, diabetes, coronary artery disease, heart failure, and chronic obstructive pulmonary disease. Figure 1 shows the design of the study. Ethics approval was granted by Griffith University.

### Participants

This trial will enrol at least 64,835 participants, sourced from the approximately 3 million Bupa Australia health fund members located across the country.

A series of inclusion and exclusion criteria have been applied prior to randomisation to reduce the risk of contamination of results from other disease management programs available to the health funds' members. Health fund members who lose eligibility through cessation of health insurance (which is estimated at less than 4% per annum based on current experience with the general insured population) will be counted only for months they have health fund membership eligibility as data will not be available thereafter.

Inclusion criteria include:

• 18-90 years of age;

• Hold hospital cover with Bupa Australia (and have for at least 12-months continuously immediately prior);

• Have a valid Australian mailing address;

• Have claims evidence of diagnosis of low back pain, diabetes, coronary artery disease, or chronic obstructive pulmonary disease based on Bupa Health Dialog proprietary identification systems that analyse participant claims history to determine presence of disease;

• Are the highest risk member of their household based on Bupa Health Dialog proprietary risk identification systems that analyse predicted risk;

• Have claims-based predicted risk levels of greater than 0.061, (which is equivalent to a predicted cost for the following 12 months of $3,050 or more, in 2010 dollars).

Exclusion Criteria will include:

• Any individual who shares a household with a previously assigned study subject;

• Individuals targeted for and/or involved in potentially similar services prior to study initiation;

• Individuals with claims evidence prior to study initiation of:

○ End Stage Renal Disease

○ HIV/AIDS

○ Haemophilia

• Individuals with claims evidence of an organ transplant prior to study initiation.

#### Randomisation and allocation concealment

Subjects are identified for enrolment each month based on qualification of inclusion criteria including a minimum risk score of 0.061. This is equivalent to a predicted cost for the following 12 months of $3,050 or more (2010 Australian dollars). Risk scores are calculated monthly and each month, a sample of subjects who meet the inclusion criteria are randomly selected using the SurveySelect procedure in SAS [17]. The proportion selected from those who meet the inclusion criteria is dependent on the expected capacity of the health coaches providing telephonic services. The samples are then randomised into the intervention or control groups. Randomisation is blinded and conducted independently through the Griffith Clinical Trials Unit

A stratified random allocation design is used to produce concordance between the intervention and control groups on baseline demographics and risk score. Randomisation was created using SurveySelect procedure using SAS [17] with the default simple random sampling option and stratified by chronic condition with a 4:1 allocation ratio to intervention and control. For each monthly batch of data, a seed value of nine digits was obtained by the statistician using random number generator with replacement from 0 to 9 for each of the nine digits. The SurveySelect procedure generates uniform random numbers using a prime modulus multiplicative generator with modulus 231 and multiplier 397204094. This means that it is very unlikely randomisation will be repeated. The effectiveness of randomisation for each monthly batch of data was checked by performing tests between the intervention and control groups on total non-maternity cost, number of overnight admitted, age, sex, and state of residential.

#### Blinding

This trial is considered to be open in design where no method has been specifically used for blinding of participants, health coaches or data collection agents.

### Interventions

#### Intervention Group

The Intervention Group will be eligible to receive disease management services from Bupa Health Dialog including mailed program awareness notifications, outbound Health Coach outreach, and follow-up calls (a minimum of one call in the first two months with no maximum number of calls being set), access on an inbound telephonic basis to Health Coaches, tailored mailed educational material, tailored outreach material designed to encourage individuals with specific risks to talk with a Health Coach, and mailed Health Coach selected educational and other material. Where English is not their first language, clients can participate by having assistance in the program from their nominated personal carer who can act as an interpreter.

#### Control Group

The control group will generally receive usual care but are given the opportunity, via mail, to "opt-in" to receive health coaching. Individuals randomly assigned to the control group will receive a letter outlining the service provided by Bupa Health Dialog. If a participant assigned to the usual support control group actively seeks health coach engagement, they will receive the same service as that provided to a participant assigned to the intervention group (i.e. health coaching). Health coaches will have the same quality of information available about the members and will have all the same educational resources at their disposal as they do for the intervention group.

All individuals assigned to the Control will be analysed as such regardless of whether they receive health coaching or not. Experience in the USA [11] suggests that the proportions of individuals in the control group who actively seek health coaching with mailed outreach only will be 3% or less. Therefore, the Control is considered to be an appropriate approximation of usual support and care. Moreover, the differentiation of service levels between the intervention and control is therefore expected to be quite high. To ensure this is the case in the Australian setting, the number of members falling into this category will be reviewed on a frequent and regular basis.

### Outcome measures

#### Primary outcome

The primary outcome will be the total (non-maternity) cost per member as reported to the private health insurer (i.e. charged to the insurer) one year post randomisation. The cost (i.e. the total benefit paid) is calculated as the sum of hospital, medical (excluding Medicare Benefits Schedule component) and prostheses claims. Ancillary benefits will not be included as the role of the intervention in impacting on a range of benefits including dental and optical is not sufficiently well defined.

#### Secondary outcomes

Secondary outcome measures will include:

• Non-maternity costs in the intervention year per member by type of service;

• Rates of inpatient (non-maternity) hospitalisations in total in the intervention year;

• Rates of "impactable inpatient hospitalisations" in the intervention year. "Impactable" inpatient hospitalisations are defined as those that are generally associated with the pre-existing condition, essentially, excluding those hospitalisations for which there could be little reasonable expectation that telephone health coaching could impact, for example, dialysis, chemotherapy for cancer, transplants;

• Rates of inpatient (non-maternity) hospitalisations by type of hospitalisation (medical, surgical, and other sub-classifications) in the intervention year;

• Same day non- maternity admission rates in the intervention year, and

• Rates of non-maternity inpatient bed days in the intervention year.

#### Other measures

Ongoing monitoring of program activity in both the intervention and control group will be regularly conducted. Individuals who engage with a health coach will be eligible to be randomly selected to participate in a satisfaction survey about their health coaching experiences.

#### Follow-up

Each study subject will be followed continuously through claims data collection until paid claims have accrued for a full 12 month period after he or she is randomised into intervention or control group. An additional four months will be allowed for the processing of claims to ensure the majority of insurance claims data have been collected within a reasonable period.

### Sample size

Sample size calculations were performed based on the coefficient of variation of the primary outcome measure of predicted total non-maternity cost for each chronic condition. A sampling ratio of 4:1 intervention group participants for every one control group participant is used to maximise the coverage for those to receive the intervention. It is expected that not all those randomised to the intervention group will be "reached" (directly contacted by telephone), and of those reached, not all will consent or engage in health coaching.

We have estimated the necessary sample size based on two assumptions; 1. at least 30% of those randomised to the intervention group will be engaged, and 2. those engaged will have a 12.5% reduction in healthcare claims over the 12-months of follow-up compared with the control group. This gives an overall expected effect size for the intervention group of 3.75%. To detect a 3.75% reduction in total non-maternity costs at the 5% level of significance with 80% power and the coefficient of variation of 2.43 (based on a sample of historical claims and observed claims in a subsequent period), an overall sample size of 64,835 is required for an intent to treat analysis.

### Data collection

#### Claims data collection

Bupa Australia claims data is accumulated as part of standard business practice. As Bupa Australia's agent, Bupa Health Dialog has legal and open access to relevant Bupa Australia claims data through a contractual relationship with Bupa Australia. Claims data will be extracted from Bupa Australia data systems.

#### Process measure data collection

Bupa Health Dialog health coaches will provide an array of data on those interactions and may also collect facts about the members to support their engagement with the members. These data will be used to provide optimal service to the participants and will be analysed to support outcome assessments on their experiences. This is independent of the trial and consistent with the health professional role of the coaches.

### Data analysis

This is an intention to treat study. The stratified randomised sampling strategy is intended to minimise baseline differences in key covariates that may influence the outcomes. Statistical testing of costs per member will be based on the natural logarithm due to the typically non-normal distribution of costs. The decision to participate in health coaching could potentially indicate differences in attitudes to prevention or compliance and so have the potential to bias outcomes. Therefore, in order to improve the precision of any test of differences between the groups, ANCOVA may be used. Covariates will include historical costs by service category, historical admission counts, age, gender, state of residence, policy type, and targeted condition flags, in as much as the resulting model is properly specified. Sub-group analysis of those with a recorded hospitalisation within 12 months prior to enrolment of the trial will be undertaken with respect to primary and secondary outcome measures. We will report the trial in accordance with the updated CONSORT statement [18].

## Discussion

The CAPICHe trial responds to the limited analyses on the effectiveness of providing chronic disease management services through telephone health coaching. It will provide an important step forward for Australian research of the effectiveness of health coaching as well as to the international debate regarding the effect of disease management programs on financial outcomes through providing an important opportunity to assess the generalisability of previous large scale trials within the United States to other populations. The perspective of private health insurers is often absent in the Australian literature, and this trial goes some way to addressing this gap. If positive, CAPICHe will present evidence that telephone health coaching service is a potentially cost-effective strategy to improve health outcomes in individuals with chronic disease in Australia.

## Abbreviations

ACTR: Australian New Zealand Clinical Trials Registry; ANCOVA: analysis of covariance; CAPICHe: Costs to Australian Private Insurance - Coaching Health; CONSORT: Consolidated Standards of Reporting Trials.

## Competing interests

Bupa Australia has contracted with Bupa Health Dialog to provide telephonic health coaching to higher risk members. Bupa Health Dialog is a fully owned and operated subsidiary of Bupa Australia.

The Bupa Health Foundation may have Board members in common with Bupa Australia.

Bupa Health Foundation has sought to contribute to the body of knowledge on the impact of telephonic coaching on chronic disease outcomes and viability, and to independently evaluate the outcomes of the intervention provided by BHD to Bupa Australia members, with the permission and encouragement of both BHD and Bupa Australia. Neither BHD nor Bupa Australia governs this study which is under the auspice of the Foundation.

## Authors' contributions

All authors meet the criteria for authorship and have read and approved the final manuscript.

## Pre-publication history

The pre-publication history for this paper can be accessed here:

http://www.biomedcentral.com/1471-2458/12/114/prepub
